# Efficient production of foot-and-mouth disease virus empty capsids in insect cells following down regulation of 3C protease activity

**DOI:** 10.1016/j.jviromet.2012.11.011

**Published:** 2013-02

**Authors:** Claudine Porta, Xiaodong Xu, Silvia Loureiro, Saravanan Paramasivam, Junyuan Ren, Tara Al-Khalil, Alison Burman, Terry Jackson, Graham J. Belsham, Stephen Curry, George P. Lomonossoff, Satya Parida, David Paton, Yanmin Li, Ginette Wilsden, Nigel Ferris, Ray Owens, Abhay Kotecha, Elizabeth Fry, David I. Stuart, Bryan Charleston, Ian M. Jones

**Affiliations:** a School of Biological Sciences, University of Reading, Reading RG6 6AJ, UK; b Institute for Animal Health, Ash Road, Pirbright, Woking GU24 0NF, UK; c Indian Veterinary Research Institute, Bangalore Campus, Hebbal, Bangalore 560024, Karnataka, India; d National Veterinary Institute, Technical University of Denmark, Lindholm, 4771 Kalvehave, Denmark; e Division of Cell & Molecular Biology, Imperial College, South Kensington, London SW7 2AZ, UK; f Department of Biological Chemistry, John Innes Centre, Norwich Research Park, Colney, Norwich NR4 7UH, UK; g Division of Structural Biology, University of Oxford, The Wellcome Trust Centre for Human Genetics, Roosevelt Drive, Oxford OX3 7BN, UK

**Keywords:** Foot-and-mouth disease virus, Recombinant baculovirus, Empty capsids, Protein processing, Frameshift, 3C protease, Vaccine

## Abstract

Foot-and-mouth disease virus (FMDV) is a significant economically and distributed globally pathogen of *Artiodactyla*. Current vaccines are chemically inactivated whole virus particles that require large-scale virus growth in strict bio-containment with the associated risks of accidental release or incomplete inactivation. Non-infectious empty capsids are structural mimics of authentic particles with no associated risk and constitute an alternate vaccine candidate. Capsids self-assemble from the processed virus structural proteins, VP0, VP3 and VP1, which are released from the structural protein precursor P1-2A by the action of the virus-encoded 3C protease. To date recombinant empty capsid assembly has been limited by poor expression levels, restricting the development of empty capsids as a viable vaccine. Here expression of the FMDV structural protein precursor P1-2A in insect cells is shown to be efficient but linkage of the cognate 3C protease to the C-terminus reduces expression significantly. Inactivation of the 3C enzyme in a P1-2A-3C cassette allows expression and intermediate levels of 3C activity resulted in efficient processing of the P1-2A precursor into the structural proteins which assembled into empty capsids. Expression was independent of the insect host cell background and leads to capsids that are recognised as authentic by a range of anti-FMDV bovine sera suggesting their feasibility as an alternate vaccine.

## Highlights

► Efficient expression of FMDV empty capsids in insect cells after moderation of 3C protease action. ► Expression cassette productive in multiple insect cell lines. ► Empty capsids visualised by transmission electron microscopy. ► Empty capsids react with wide range of positive sera as well as authentic virus. ► Efficient empty capsid synthesis may allow development as a vaccine.

## 1 Introduction

Foot-and-mouth disease virus (FMDV) is the prototypic aphthovirus within the family *Picornaviridae* (reviewed by (Grubman and Baxt, 2004)). Economic losses from foot-and-mouth disease outbreaks are among the highest of all livestock diseases and widespread vaccination is the method of choice for disease control (Rodriguez and Grubman, 2009). The current vaccine is a killed whole virus vaccine whose limitations have been widely discussed; growth of the live virus prior to inactivation is not without risk, the appropriate serotype for each outbreak is required, some field strains grow poorly before adaptation and immunogenicity can be lost upon storage (Rodriguez and Grubman, 2009). To address these issues alternate vaccines have been sought, among them the use of empty capsids which are structural and immunogenic mimics of virus particles but lack the potential for causing disease outbreak (Rowlands et al., 1975; Rweyemamu et al., 1979).

As for all picornaviruses, the icosahedral FMDV capsid is assembled from mature proteins derived from a structural precursor, P1-2A, following cleavage *in trans* by the 3C protease (reviewed by (Belsham, 2005)). The 3C protease is one of many non-structural proteins synthesised in the infected cell but expression of P1-2A and 3C in the absence of any other FMDV encoded protein in recombinant systems is sufficient to afford authentic precursor cleavage (Lewis et al., 1991; Roosien et al., 1990). Accordingly there have been a number of reports of the assembly of recombinant FMDV empty capsids following the use of expression systems such as vaccinia virus (Abrams et al., 1995), adenovirus (Mason et al., 2003), *E. coli* (Cao et al., 2010; Lewis et al., 1991), transgenic plants (Pan et al., 2008) and baculovirus (Li et al., 2008; Oem et al., 2007; Roosien et al., 1990). In some cases, sufficient empty capsid material has been prepared to immunise cattle and protection against homologous challenge was demonstrated (Li et al., 2008, 2011) but in the main the configuration of the P1and 3C coding sequences used to achieve empty capsid expression and the efficiency of capsid assembly has been highly variable, particularly in insect cells. For example, using the successful expression of swine vesicular disease empty capsids as an exemplar (Ko et al., 2005), usage of a dual promoter vector in which the P1 and 3C coding sequences of an O serotype of FMDV were under the control of the baculovirus polyhedrin and p10 promoters respectively, resulted in incomplete precursor cleavage and predominantly pentameric assemblies rather than complete capsids (Oem et al., 2007). Similarly, a dual expression strategy of an Asia 1 serotype of FMDV led to incomplete cleavage of the P1-2A precursor (Cao et al., 2009). More recently, forsaking the use of 3C to generate the mature capsid proteins, VP0 and VP3-2A-VP1 from an O serotype of FMDV were co-expressed relying on self-cleavage at the 2A site (Donnelly et al., 2001) to generate the requisite structural proteins for assembly, which resulted in partial success (Cao et al., 2010). In a further example, a Bombyx (silk worm) baculovirus system encoding a P1-2A-3C sequence of an FMDV Asia 1 isolate was used as a single transcription unit driven by the polyhedrin promoter and the empty capsid material harvested from the haemolymph of the infected silk worms was immunogenic in cattle and led to levels of neutralising antibody associated with protection (Li et al., 2008, 2011). Despite these successes, variation associated with both the FMDV serotype and the host cell background used mean that a uniform genetic design capable of producing empty capsids for any serotype has yet to be reported. Recently, the yield of empty capsids from insect cells for another picornavirus, human enterovirus 71, was improved by use of a dual vector in which a less active promoter, the CMV early promoter, was used for transcription of the 3C coding unit whilst the strong polyhedrin promoter directed expression of the P1 structural precursor (Chung et al., 2010). However, yield improvement was restricted to Sf9 cells as infection of T.ni cells, an alternative insect cell line that generally gives higher expression levels (Davis et al., 1993), resulted in poor capsid expression plausibly as a result of low levels of promoter-specific transcription factors present in T.ni cells. Picornaviruses traditionally exhibit a strong “host cell shutoff” phenotype which is partly the result of 3C protease cleavage of host cell proteins in addition to its action on the virus structural precursor P1 (Li et al., 2001; Strong and Belsham, 2004). As a result, picornavirus replication cycles are typically rapid and exhibit extensive cytopathic effect (*e.g.* (Rodriguez Pulido et al., 2007)). It follows that if cleavage of host cell proteins by FMDV 3C protease was to occur in a recombinant empty capsid expression system it would curtail the expression period and limit the yield of capsids observed. Thus, variable levels of 3C expression could account for the variation in the levels of FMDV empty capsids reported to date and purposeful moderation of 3C activity might enhance the yield of capsid observed, allowing a more thorough exploration of their virtues as vaccine candidates. Here a new genetic design is described for the expression of empty FMDV capsids in insect cells following infection by recombinant baculoviruses expressing P1-2A-3C with a number of modifications to reduce 3C activity.

## 2 Materials and methods

### 2.1 Cell culture and virus growth

Sf9, T.ni and T.nao38 cells were cultured in BioWhittaker^®^Insect-Xpress (Lonza, Basel, Switzerland) supplemented with 2% FCS, 100 units/ml penicillin, 100 μg/ml streptomycin and 2.5 μg/ml amphotericin B. Cells were grown at 28 °C as monolayers or in suspension with agitation at 100 rpm. Baculoviruses were generally amplified in monolayer cultures but large scale infections for capsid isolation were done in suspension. Virus stocks were titreed using plaque assay on Sf9 monolayers.

### 2.2 Sequences and cloning

The sequence for FMDV A22 Iraq (AY593764.1) was that deposited in the database. DNA was synthesised *de novo* (Lifetechnologies, Carlsbad, USA). The transfer vector used for all expressions was based on pOPINE (Berrow et al., 2007), itself a derivative of pTriEx1.1 (EMD Biosciences, Billerica, USA) and a fragment encoding P1-2A-3C of FMDV serotype A22 was cloned downstream of the p10 promoter by use of In-Fusion Technology (Clontech, Mountain View, USA). Mutations within this cassette were introduced by the swapping of appropriate fragments *via* unique restriction sites introduced during gene synthesis. Recombination between pOPINE vectors and AcMNPV bacmid KO1629 in insect cells was as described (Zhao et al., 2003). Routine DNA procedures *in vitro* made use of standard protocols or, when kits were used, those recommended by the vendor. All vectors were confirmed by DNA sequencing prior to use for expression.

### 2.3 Electrophoresis and Western blotting

Protein samples were separated on pre-cast 10% Tris–HCl SDS-polyacrylamide gels (BioRad, Berkeley, USA) and transferred to Immobilon-P membranes (Millipore, Billerica, USA) using a semi-dry blotter. For 10-well gels each sample loaded represented 5 × 10^4^ cells. Following transfer, filters were blocked for 1 h at room temperature using PBS containing 0.1% v/v Tween-20 (PBS-T), 5% w/v milk powder. Primary antibodies were used at 1 μg/ml or at a dilution of 1:1000 in PBS-T, 5% w/v milk powder. Following several washes with PBS-T, the membranes were incubated for 1 h with the appropriate HRP-conjugates and the bound antibodies detected by BM chemiluminescence (Roche, Basel, Switzerland).

### 2.4 Purification of empty capsids

Infected cultures (typically ∼10^9^ cells) were harvested at 3 days pi. and lysed by resuspension in 1/20th of the original volume of 1% Triton X-100 in PBS and held at 4 °C for 30 min with occasional agitation. Unbroken cells and nuclei were removed by centrifugation (4500 rpm, 15 min) and the clarified lysate layered onto a 30% sucrose (w/v in PBS) cushion. After centrifugation at 100,000 × *g* for 100 min the supernatant was discarded and the pellet resuspended in 1/10 tube volume of PBS containing 3500 units of benzonase. After 30 min at room temperature, the solution was clarified and applied to a preformed discontinuous 30–60% sucrose (w/v in PBS) gradient made up by seven increments of 5%. The gradient was developed by centrifugation at 100,000 × *g* for 16 h and fractionated from the top.

### 2.5 Assessing capsid antigenicity

Assays to measure the reactivity of recombinant empty capsids with a range of FMDV serotype specific polyclonal antisera were performed in a competitive ELISA format as described (Li et al., 2012). Data were expressed as percentage inhibition scores compared to an OIE standard antigen preparation. Statistical significance was assessed by one-way analysis of variance (ANOVA).

### 2.6 Electron microscopy

Samples were allowed to adhere to carbon coated formvar grids for 5 min at room temperature followed by a brief water wash (1 min) before staining with 1% uranyl acetate for 1 min. Excess stain was removed by blotting and the grids examined on a Philips CM20 (Philips Electronics, Amsterdam, Netherlands) operating at 80 kV.

## 3 Results

**Fig. 1:**
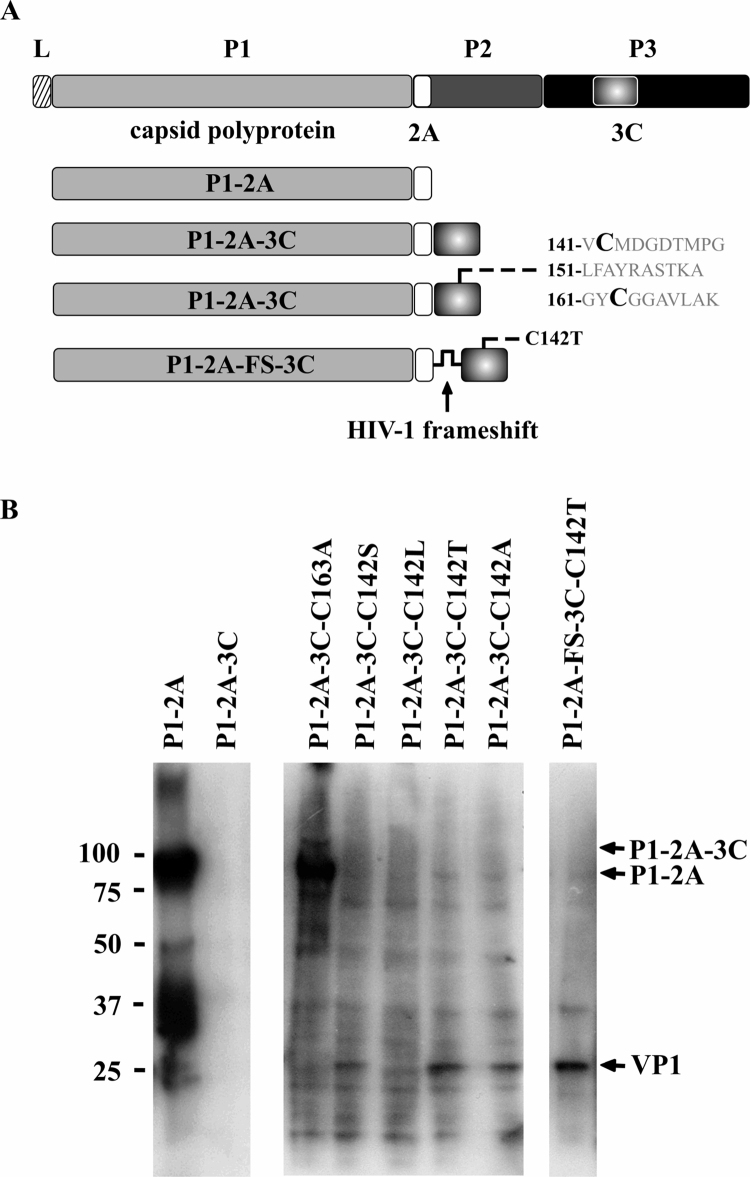
Expression screening of various FMDV P1-2A ± 3C cassettes. (A) Cartoon representation of the genetic designs tested. The text near the bottom right represents the amino acid sequence of FMDV 3C between residues 141–170 with Cys 142 and Cys 163 highlighted. (B) Outcome of screening the various A22 Iraq constructs by immunoblotting using a polyvalent A serum. The expected migration positions of P1-2A-3C, P1-2A and VP1 are indicated although very little P1-2A-3C is visible. Numbers to the left are the migration positions of protein markers and are in kilodaltons.

**Fig. 2:**
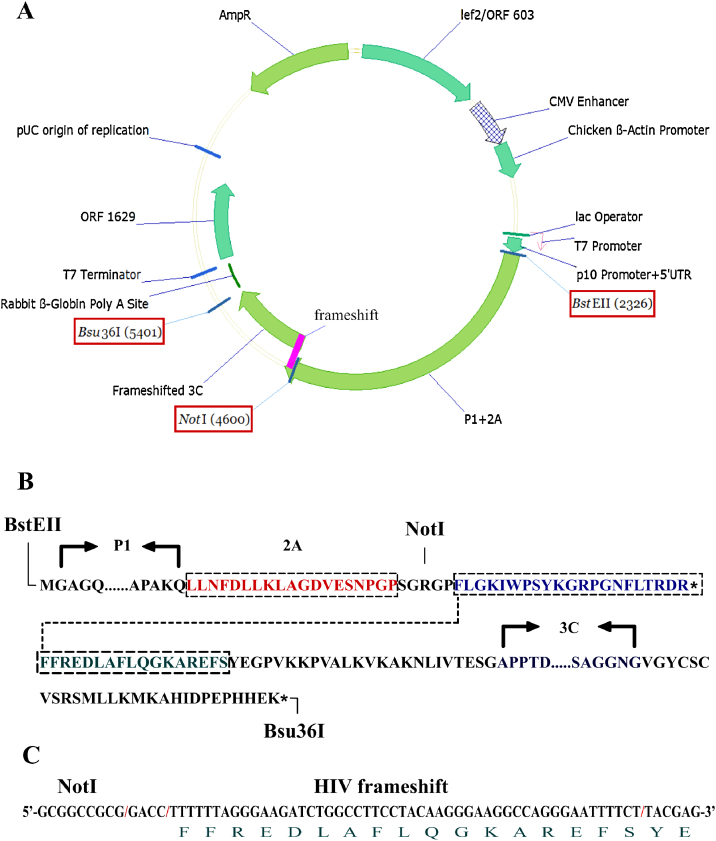
Cartoon representation of the vector used for expression of empty FMDV capsids with the salient features shown. Unique restriction sites available for the exchange of the capsid polyprotein precursor coding region from different serotypes are indicated by red boxes. (B) Translated ORF details of the P1-2A polyprotein, the 3C protease and the junctions between them. The frameshift event, which begins on the overlapping Phe residue and results in a single polyprotein linking the P1-2A and 3C proteins, is indicated by the dotted line. (C) Sequence detail of the HIV frameshift (above) and its relationship to the translated product (below).

Preliminary experiments with constructs (Fig. 1A) encoding the previously defined minimum proteins for FMDV capsid protein expression and cleavage (Lewis et al., 1991; Roosien et al., 1990) based on a synthetic sequence of FMDV A22 Iraq revealed that expression of P1-2A led to the abundant synthesis of FMDV-related antigen of the expected molecular weight (∼90 kDa) as well as some non-specific degradation products, whilst fusion of 3C in phase with the C terminus of P1-2A effectively abolished expression (Fig. 1B). When the same expression cassette included a site-directed change of the 3C active site cysteine 163 to alanine (Sweeney et al., 2007), expression of a protein of the molecular weight of P1-2A fused to 3C (a minor product) in addition to P1-2A (the predominant product) (∼110 kDa and ∼90 kDa, respectively) was observed (Fig. 1B). Thus, 3C activity and not a *cis* acting sequence acts to restrict P1-2A expression. As a lower 3C activity might be compatible with an enzyme level sufficient to allow P1-2A expression and subsequent cleavage, expression cassettes were designed to reduce 3C activity. Two approaches were used: site-directed mutagenesis of 3C, based on the three-dimensional structure of the enzyme, that had previously been shown to reduce enzyme activity *in vitro* (Sweeney et al., 2007), and the introduction of the HIV-1 ribosomal frameshift site, which has been shown to function in insect cells (Adamson et al., 2003), between the P1-2A and 3C coding sequences (Fig. 1A). Expression screening revealed that individual changes at 3C residue 142 led to the detection of a band at ∼26 kDa consistent with the expected molecular weight of the mature VP1 product following 3C cleavage of the P1-2A precursor protein. A combination of both the frameshift and the 3C Cys142Thr mutation was the most productive approach leading to almost complete processing of P1-2A as monitored by the appearance of VP1 (Fig. 1B). The serum used for the Western blot detection of FMDV proteins was polyvalent but reactivity was largely directed to epitopes in VP1. However, some reactivity with intermediates (*e.g.* VP3-VP1) in the cleavage reaction is apparent. In addition, the resulting optimised vector allows exchange of the FMDV P1-2A coding region *via* unique restriction sites (Fig. 2A) permitting any FMDV capsid serotype to be juxtaposed to the moderated 3C translation product (Fig. 2B).

**Fig. 3:**
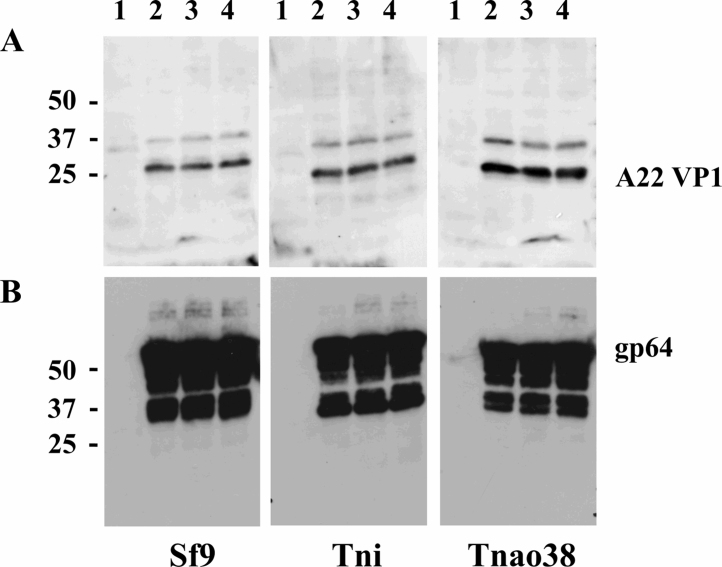
Time course of expression of FMDV A22 antigen in three insect cell lines. Numbers above the track are days post infection in each of the cell lines indicated, with the exception of track 1 which is the mock infected control. In panel A, the serum used for immunodetection was a Guinea pig polyvalent A serotype (a reagent of the Institute for Animal Health). Panel B is an infection control using a monoclonal antibody to the baculovirus surface glycoprotein gp64. Numbers to the left are the migration position of protein markers and are in kilodaltons.

A feature of the expression design described here, which differs from most previous examples, is that only one transcription unit is present, in this case driven by the AcMNPV p10 promoter and control of 3C expression is achieved wholly at the level of translation. Previous examples of high level picornavirus expression with moderated 3C activity have used different promoters but exhibited strong host cell variability (Chung et al., 2010). To assess expression in a variety of insect cells, a high titre baculovirus stock of the optimal A22 P1-2A-FS-3C-C142T construct was used to infect *Spodoptera frugiperda* (Sf9) and two *Trichoplusia ni* (T.ni and T.nao38) cell lines and a time course of FMDV antigen expression was conducted. Sf9 cells are widely used for recombinant baculovirus work but T.ni lines have been shown to give higher expression levels in some cases (Hashimoto et al., 2010, 2012; Maruniak et al., 1994). FMDV VP1 antigen was apparent at 2 days post infection in all lines tested with peak levels occurring at 3 and 4 days post infection (Fig. 3A) typical of baculovirus very late promoter driven expression (Loureiro et al., 2011; Pengelley et al., 2006). Little unprocessed P1-2A was observed suggesting that expression and cleavage are tightly coupled, as expected of a polyprotein and protease translated from a single mRNA. Both T.ni cell lines expressed higher levels of FMDV antigen when compared to Sf9 cells on a volume equivalent basis with the rank order, based on VP1 band intensity, being T.nao38 > T.ni > >Sf9. Differences in signal intensity in the different cell lines did not relate to levels of baculovirus infection as Western blotting for a baculovirus encoded protein, the major structural glycoprotein gp64, showed similar levels of infection (Fig. 3B). In addition, one commercial cell line, VE cells, in which a viral ankaryn repeat protein from *Campoletis sonorensis* ichnovirus has been integrated into the Sf9 genome to reduce levels of AcMPNV-induced apoptosis (Fath-Goodin et al., 2009) was tested but they offered no advantage when compared to unmodified Sf9 cells (unpublished observations). Based on the results obtained analogous constructs for the seven FMDV serotypes should express similarly although further engineering may be required to optimise the expression level of any one sequence.

**Fig. 4:**
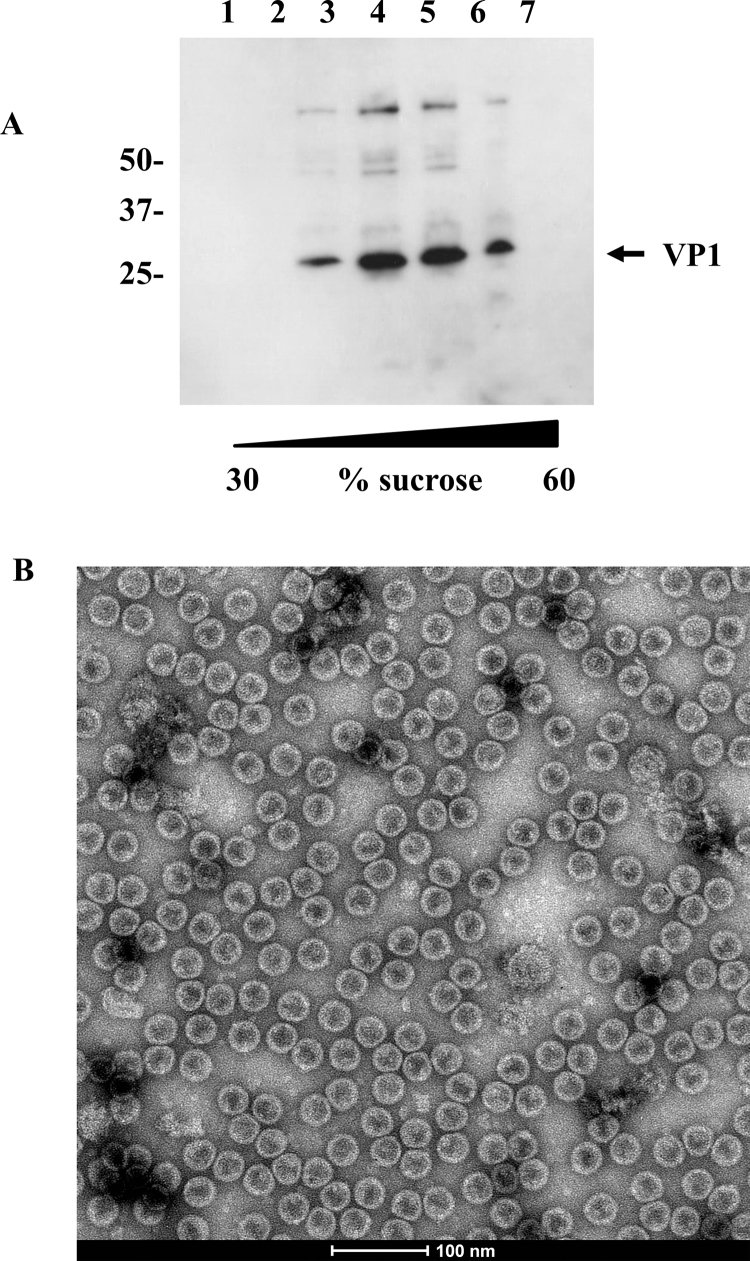
Analysis of empty capsid assembly. (A) Sucrose gradient fractionation of FMDV capsid material analysed by Western blot using A22 serotype antisera with an extract of A22 recombinant virus infectedSf9 cells. Numbers at the top are fractions numbers. Numbers to the left are the migration position of protein markers and are in kilodaltons. (B) Peak fractions from the gradient were analysed by TEM. The bar is 100 nm.

Cleavage of the FMDV P1-2A precursor protein into mature structural proteins can lead to empty capsid assembly (Cao et al., 2009, 2010) although incomplete assembly has also been observed (Oem et al., 2007). To test for the production of empty capsids in the system described here, extracts of infected insect cells harvested at 3 days post infection were clarified by low speed centrifugation and the particulate material present in the supernatant was collected by sedimentation through a 30% sucrose cushion and subsequently on a sucrose velocity gradient. Fractions from the middle of the gradient (∼40% sucrose), typically the sedimentation position of intact empty capsids, were positive for VP1 by Western blot (Fig. 4A). Electron microscopy of the same fractions revealed uniform structures typical of picornavirus capsids with stain penetration consistent with empty capsid assembly (Fig. 4B).

**Fig. 5:**
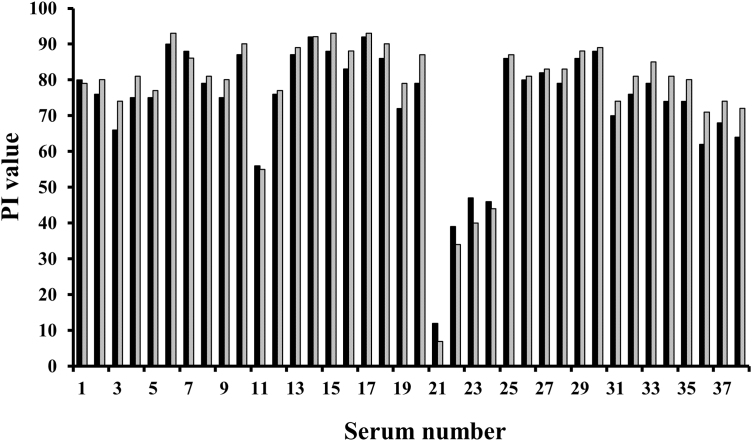
Antigenic characterisation of recombinant FMDV capsids. Comparison of serotype A22 Iraq native antigen and recombinant A22 empty capsids (solid/grey bars) in a twin site ELISA with a panel of serum samples. The sera are from animals vaccinated with A Iran05 (1–10), vaccinated and challenged with A24 Cruzeiro (11, 12), vaccinated and challenged with A Iran96 (13–18), infected with A22 Iraq 24/64 (19, 20), infected with A24 Cruzeiro (21–24), infected with A Malaysia 97 (25, 26), infected with A Iran99 (27, 28) and vaccinated with A Iran05 (29–38). PI – percentage inhibition.

To ensure the empty capsids observed were authentic structural mimics of FMDV particles they were compared with inactivated FMDV grown in BHK21 cells in a sandwich ELISA format using a number of interrogating sera (Li et al., 2012). Of 38 different bovine sera, representing seroconversion to infection or vaccination with serotype A virus or vaccine, no significant differences (*P* < 0.001) were observed between the performance of the recombinant empty capsids and the more established virus antigen source (Fig. 5). These data suggest that the recombinant empty capsids are faithful mimics of authentic virus, at least to the level probed by the antibodies used here. Purified empty FMDV capsids expressed in insect cells raised neutralising antibodies in guinea-pigs as measured by two OIE prescribed tests for the presence of specific antibodies: the virus neutralisation test (VNT) and the liquid phase blocking ELISA (LPBE) with the levels of neutralising antibody consistent with protection in animal models (not shown).

## 4 Discussion

A new genetic design has been investigated as a robust platform technology for the efficient production of empty FMDV capsids. Previous approaches to recombinant picornavirus particle synthesis in insect cells have included the expression of the complete picornaviruses genomes (Brautigam et al., 1993; Rosen et al., 1993) as well as co-expression of only the structural precursor protein P1 (or P1-2A) and protease, but the levels of capsid obtained have been variable (Cao et al., 2010; Ko et al., 2005; Li et al., 2008, 2011; Oem et al., 2007; Roosien et al., 1990). A systematic analysis of the causes of the observed variation is not available although the lower pH of insect cell media is not a major factor (Medina et al., 1995). FMDV 3C enzymes have a reported wide range of cellular targets in addition to the virus P1-2A structural protein precursor suggesting that host protein cleavage could occur in insect cells to effectively curtail expression over the 2–4 days of a baculovirus infection. A reduced level of 3C was anticipated to be sufficient to enable P1-2A cleavage but be insufficient to cause general toxicity. Bicistronic vectors, in which the 3C coding sequence is transcribed from a less active promoter have been encouraging but were dependent on host cell factors (Chung et al., 2010) suggesting that other methods of translational control, for example through the use of IRES or frameshift elements (Probst, 2002; Royall et al., 2004) might be usefully applied. Combining structure-based amino acid substitutions in a flap that folds over the substrate binding pocket of 3C (Sweeney et al., 2007; Zunszain et al., 2010), with the introduction of a frameshift signal between the P1-2A and 3C coding regions designed to lower the level of 3C translated rescued capsid protein expression. The well characterised HIV-1 frameshift (Dinman, 2012) has a measured -1 shift rate of about ∼1:20 in mammalian cells suggesting 3C levels would be reduced to 5% of those associated with the in-frame P1-2A-3C cassette. However, as the rate of frameshift in insect cells has not been measured directly, the actual amount of 3C expression remains to be determined. It is noteworthy that the β-ribbon modified here was also observed in other recent 3C enzyme structures (Cui et al., 2011; Lu et al., 2011; Norder et al., 2011) indicating that similar strategies to reduction in 3C activity may be usefully applied to the synthesis of other picornavirus empty capsids. Similarly, frameshifting rates may be altered through modification of the “slippery sequence” on which the ribosome shifts register (Wilson et al., 1988), suggesting further optimisation may be feasible. The expression strategy shown here for the FMDV A serotype has also been successful for O and SAT2 serotypes in preliminary experiments (not shown) implying that the technology may be generally applicable to all serotypes and offer a choice of cell line for scale-up. Empty FMDV capsids were visually uniform by TEM and antigenically indistinguishable from authentic virus in twin site ELISA. Empty capsids are an attractive vaccine candidate for FMDV not only because they do not require high biological containment facilities but also because they allow modification to capsid sequences that would render infectious virus nonviable. Thus capsid engineering, for example for improved stability, broadened immune response (Tobin et al., 2008) or DIVA (Differentiating Infected from Vaccinated Animals) compliance may be feasible using the technology described.
